# Chitosan Improves Anti-Biofilm Efficacy of Gentamicin through Facilitating Antibiotic Penetration

**DOI:** 10.3390/ijms151222296

**Published:** 2014-12-03

**Authors:** Haibo Mu, Fan Guo, Hong Niu, Qianjin Liu, Shunchun Wang, Jinyou Duan

**Affiliations:** 1 College of Science, Northwest A&F University, Yangling 712100, China; E-Mails: mhb1025@nwsuaf.edu.cn (H.M.); amin@nwsuaf.edu.cn (F.G.); dawn@nwsuaf.edu.cn (H.N.); 2011015288@nwsuaf.edu.cn (Q.L.); 2 The MOE Key Laboratory for Standardization of Chinese Medicines, Institute of Chinese Materia Medica, Shanghai University of Traditional Chinese Medicine, 1200 Cailun Road, Shanghai 201203, China

**Keywords:** chitosan, antibiotic, biofilms, penetration

## Abstract

Antibiotic overuse is one of the major drivers in the generation of antibiotic resistant “super bugs” that can potentially cause serious effects on health. In this study, we reported that the polycationic polysaccharide, chitosan could improve the efficacy of a given antibiotic (gentamicin) to combat bacterial biofilms, the universal lifestyle of microbes in the world. Short- or long-term treatment with the mixture of chitosan and gentamicin resulted in the dispersal of *Listeria monocytogenes* (*L. monocytogenes*) biofilms. In this combination, chitosan with a moderate molecular mass (~13 kDa) and high *N*-deacetylation degree (~88% DD) elicited an optimal anti-biofilm and bactericidal activity. Mechanistic insights indicated that chitosan facilitated the entry of gentamicin into the architecture of *L. monocytogenes* biofilms. Finally, we showed that this combination was also effective in the eradication of biofilms built by two other *Listeria* species, *Listeria welshimeri* and *Listeria innocua.* Thus, our findings pointed out that chitosan supplementation might overcome the resistance of *Listeria* biofilms to gentamicin, which might be helpful in prevention of gentamicin overuse in case of combating *Listeria* biofilms when this specific antibiotic was recommended.

## 1. Introduction

Microbial biofilms are communities of sessile microorganisms embedded in a hydrated matrix of extracellular polymeric substances [1]. Microbial biofilms have been implicated in >80% of human infections such as periodontitis, urethritis, endocarditis, and device-associated infections [2]. Because of their resistance to a wide range of antibiotics, biofilm bacteria are a major concern for clinicians in the treatment of infections [3]. The emergence of resistant bacteria to conventional antimicrobials clearly shows that new biofilm control strategies are required [4].

Chitosan is the *N*-deacetylated derivative of chitin, a naturally abundant mucopolysaccharide consisting of 2-acetamido-2-deoxy-*co*-d-glucose. Chitosan has been shown to be useful in many different areas as an antimicrobial compound, as a potential elicitor of plant defense responses, as a flocculating agent in wastewater treatment, as an additive in the food industry, as a hydrating agent in cosmetics, and more recently as a pharmaceutical agent in biomedicine [5]. In this context, the antimicrobial activity of chitosan and its derivatives against different groups of microorganisms has received considerable attention in recent years [6]. Chitosan has several advantages over other types of disinfectants because it possesses a higher antibacterial activity, a broader spectrum of activity, a higher killing rate, and a lower toxicity toward mammalian cells. Also, chitosan exhibits anti-biofilm activities and the ability of chitosan to damage biofilms formed by microbes has been documented [7].

Previously, we have reported a synergistic effect between streptomycin and chitosan on the disruption of *Listeria monocytogenes* biofilms [8]. In the current study, we surveyed the synergism between chitosan and four different classes of antibiotics on the removal of *Listeria* biofilms. We found that chitosan could improve anti-biofilm efficacy of gentamicin belonging to the aminoglycoside family against *Listeria* biofilms.

## 2. Results and Discussion

### 2.1. Chitosan Promoted Gentamicin-Induced Dispersal of Listeria Monocytogenes Biofilms

*Listeria monocytogenes* is one of the most important causative agents of the serious foodborne disease, listeriosis in a wide range of mammalians [9,10,11]. *L. monocytogenes* may produce multicellular biofilms most frequently in food-related environments where bacterial cells have been shown to be much more resistant to stress and to sanitizing agents than planktonic cells [12]. To see whether chitosan and antibiotics had a synergistic effect on dispersal of *L. monocytogenes* biofilms, four classes of antibiotics that were widely used and represented different antibiotic families were initially chosen. They included gentamicin (belonging to the aminoglycoside family), rifampicin (belonging to the naphthalenic ansamycin family), tetracycline (belonging to the tetracycline family) and carbenicillin (belonging to the penicillin family).

**Figure 1:**
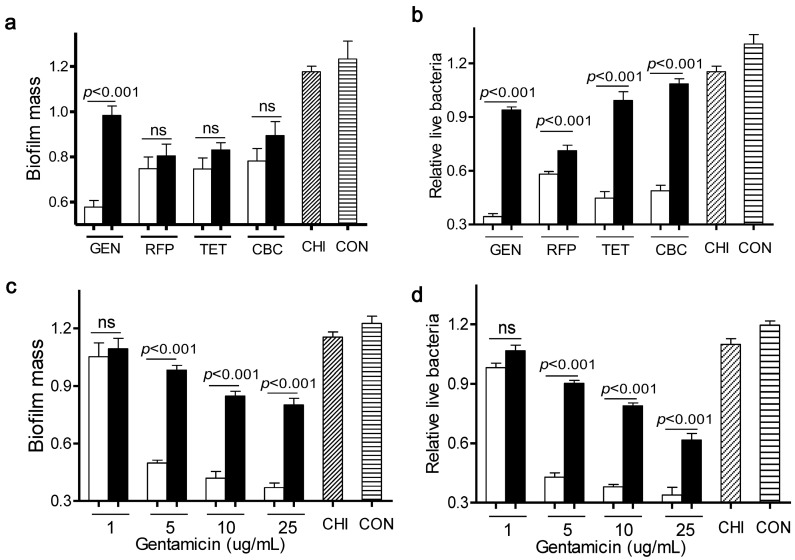
Different effects of antibiotics in combination with chitosan against *L. monocytogenes* biofilms. (**a**,**b**) Biofilms were exposed to chitosan (CHI, 200 µg/mL), different antibiotics (gentamicin (GEN), rifampicin (RFP), tetracycline (TET) and carbenicillin (CBC), 5 µg/mL) or the respective mixtures for 24 h; (**c**,**d**) Biofilms were exposed to chitosan (200 µg/mL), different concentrations of gentamicin (1, 5, 10, 25 µg/mL) or the respective mixtures for 24 h. Biofilms incubated with tryptic soy broth (TSB) were used as control. Biofilm mass and viable cells were quantified. White column: chitosan-antibiotic combination; Black column: antibiotic alone; Column with italic lines: Chitosan alone; Column with horizontal lines: control. Error bars represent SD. ns: no significant difference.

As shown in Figure 1a,b, four antibiotics at 5 µg/mL elicited a very mild capacity in dispersal of *L. monocytogenes* biofilms and killing of live bacteria, compared to the control. Meanwhile, chitosan alone at 200 µg/mL had little effect on both bioflim mass and viable cells. In contrast, when *L. monocytogenes* biofilms were exposed to a mixture of chitosan and individual antibiotic, there were much fewer viable cells than with the antibiotic alone. This observation was consistent with the concept that the chitosan combination could enhance the antimicrobial activity of antibiotics [13,14]. Interestingly, only a combination of gentamicin and chitosan significantly dispersed *L. monocytogenes* biofilms, when compared to the individual antibiotic alone. These data indicated that chitosan selectively increased the anti-biofilm efficacy of particular antibiotics such as gentamicin.

To further explore the synergism between chitosan and gentamicin in biofilm dispersal, we treated *L. monocytogenes* biofilms for 24 h with chitosan (200 µg/mL) in the presence of various concentrations of gentamicin (1, 5, 10, 25 µg/mL). Results showed that a sufficient amount of gentamicin was required for this combination to remain the anti-biofilm and bactericidal activity towards *L. monocytogenes* biofilms (Figure 1c,d). Then, we determined the synergistic interactions between gentamicin and chitosan using Check board titration [15]. As shown in Table 1, gentamicin showed a clear synergistic action with chitosan against *L. monocytogenes* biofilms (Fractional inhibitory concentration index (FIC_index_) <0.15).

**Table 1:** Fractional inhibitory concentration index (FIC_index_) of combination of gentamicin with chitosan against *L. monocytogenes* biofilms.

EC_50_ (µg/mL)				FIC_index_	Remark
Alone		Combination			
GEN	CHI	GEN	CHI		
50	>4000	5	200	<0.15	Synergy

EC_50_ is defined as the concentration of compounds required to disperse 50% *L. monocytogenes* biofilm; Synergism by the checkerboard method was defined as a FIC_index_ ≤ 0.5.

**Figure 2:**
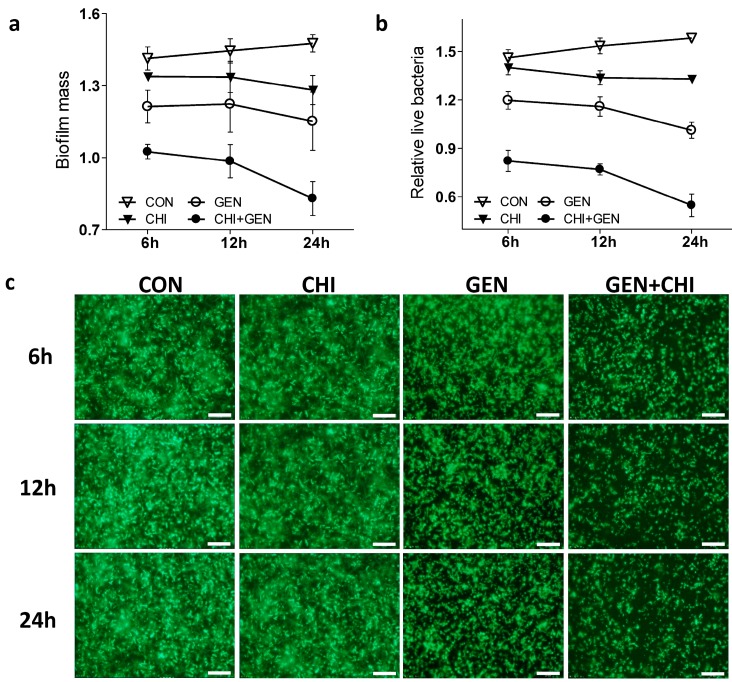
The gentamicin/chitosan combination disrupted the *L. monocytogenes* biofilms in a time-dependent manner. Biofilms were exposed to chitosan (200 µg/mL), gentamicin (5 µg/mL) or the mixture for 6, 12 and 24 h. Biofilms incubated with TSB were used as control (CON). Biofilm mass (**a**) and viable cells (**b**) were quantified, and biofilm architectures were further examined by fluorescence microscopy (**c**). Scale bars were 10 µm.

**Figure 3:**
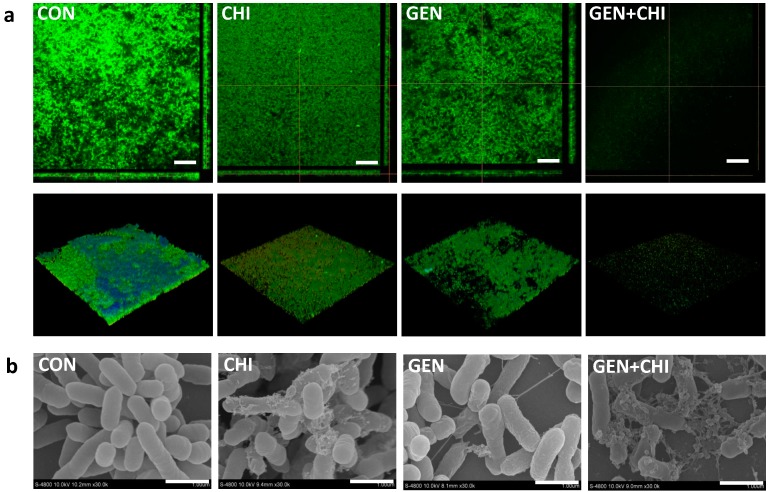
Disruption of biofilm architectures by the gentamicin/chitosan combination. *L. monocytogenes* biofilms were exposed to chitosan (200 µg/mL), gentamicin (5 µg/mL) or the mixture for 24 h. Biofilms incubated with TSB were used as control. (**a**) Confocal microscopy, three-dimensional reconstructions were shown in the bottom views, scale bars were 50 µm; (**b**) Scanning electron microscopy, scale bars were 1 µm.

Next we tested whether the gentamicin/chitosan combination dispersed *L. monocytogenes* biofilms in a time-dependent fashion. As shown, this combination demonstrated a more pronounced effect in the decrease of both biofilm mass (Figure 2a) and viable cells (Figure 2b) than gentamicin or chitosan alone after short- or long-term treatment. These observations were also evidenced by visualization of biofilms by fluorescence microscopy (Figure 2c), confocal microscopy (Figure 3a) and scanning electron microscopy (Figure 3b), in which there were fewer scattered cell aggregates in *L. monocytogenes* biofilms after exposure to the gentamicin/chitosan combination than that of individual reagents.

### 2.2. Factors Involved in Anti-Biofilm Capacity of the Gentamicin/Chitosan Combination

**Figure 4:**
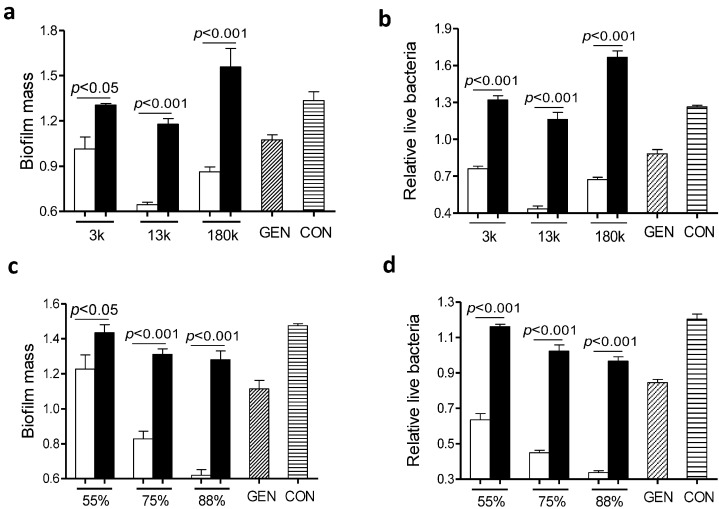
Chitosan with specific molecular mass and *N*-deacetylation degree was essential in retaining the high anti-biofilm capacity of the gentamicin/chitosan combination. *L. monocytogenes* biofilms were exposed to chitosan (200 µg/mL), gentamicin (5 µg/mL) or the mixture for 24 h. (**a**,**b**) Chitosan with different molecular mass (3000, 13,000 and 180,000 Da); (**c**,**d**) Chitosan with different *N*-deacetylation degrees (DD: 50%, 75% and 88%). White column: chitosan-antibiotic combination; Black column: chitosan alone; Column with italic lines: antibiotic alone; Column with horizontal lines: control. Error bars represent SD.

It is generally accepted that the anti-biofilm effect of chitosan is largely dependent on *N*-deacetylation degrees [6] and molecular weights [16]. To examine whether the molecular mass of chitosan played a role in the anti-biofilm capacity of the gentamicin/chitosan combination, *L. monocytogenes* biofilms were exposed to gentamicin in the presence of chitosan with different molecular mass of ~3000, ~13,000 or 180,000 Da. All three chitosan-gentamicin mixtures tested reduced more biofilm mass than the respective chitosan alone did (Figure 4a,b). In addition, a combination of gentamicin and chitosan with a molecular mass of ~13,000 Da elicited the highest anti-biofilm activity of all experimental groups.

**Figure 5:**
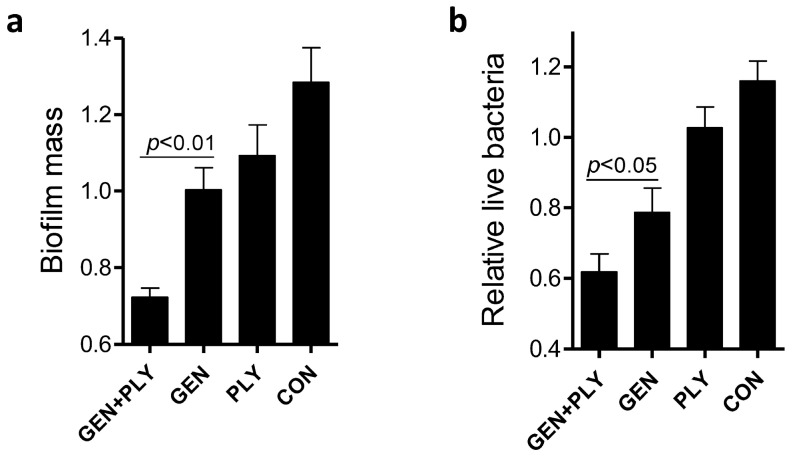
Effects of the poly-l-lysine/gentamicin combination on disruption of *L. monocytogenes* biofilms. *L. monocytogenes* biofilms were exposed to poly-l-lysine (PLY, 200 µg/mL), gentamicin (5 µg/mL) or the respective mixture for 24 h. Biofilm mass (**a**) and viable cells (**b**) were quantified. Error bars represent SD.

Next we evaluated the impact of positive charges of chitosan on the anti-biofilm capacity of the gentamicin/chitosan combinations. Chitosan with different *N*-deacetylation degrees (DD: 50%, 75%, 88%) alone was very limited in disruption of biofilms (Figure 4c,d). Although all three chitosan-gentamicin mixtures tested reduced more biofilm mass than the respective chitosan alone did, it was obvious that gentamicin combined with chitosan possessing the highest *N*-deacetylation degree displayed a stronger anti-biofilm activity. This raised the question whether other antibacterial polycationic biopolymers such as poly-l-lysine [17], instead of chitosan, also worked in a similar fashion. Results showed that the combination of poly-l-lysine and gentamicin indeed had stronger anti-biofilm and bactericidal activities than the individual agent alone did (Figure 5). This finding suggests that polycationic properties enabled higher anti-biofilm and bactericidal capacities for biopolymer-antibiotic combinations.

### 2.3. Effects of the Gentamicin/Chitosan Combination on Different Stages of L. monocytogenes Biofilms

**Figure 6:**
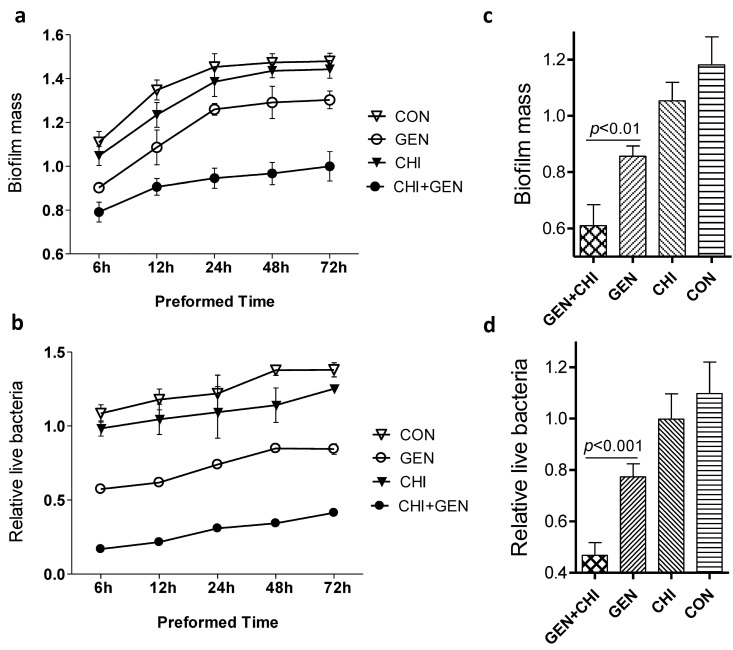
Effects of the gentamicin/chitosan combination on different stages of *L. monocytogenes* biofilms. (**a**,**b**) Biofilms were preformed for 6, 12, 24, 48 and 72 h, and then exposed to chitosan (200 µg/mL), gentamicin (5 µg/mL) or the mixture for 24 h; (**c**,**d**) *L. monocytogenes* were seeded in 96-well plates in the presence of chitosan (200 µg/mL), gentamicin (5 µg/mL) or the respective mixture for 24 h. Biofilm mass and viable cells were quantified. Error bars represent SD.

To ask whether the anti-biofilm of the gentamicin/chitosan combination depended on different stages of biofilm formation, planktonic *L. monocytogenes* were seeded in 96-well plates for 6, 12, 24, 48 or 72 h and then treated for 24 h with individual reagents or their combination. At all time-points tested, the gentamicin/chitosan combination displayed a stronger anti-biofilm and bactericidal activity than individual reagent alone did (Figure 6a,b). A similar finding was also observed in the case of planktonic *L. monocytogenes* exposed to reagents at the beginning (Figure 6c,d). These data clearly indicate that the gentamicin-chitosan combination was effective in the inhibition of biofilm formation and disruption of immature or mature biofilms at different stages.

### 2.4. Mechanistic Insights into the Anti-Biofilm Capability of the Gentamicin/Chitosan Combination

**Figure 7:**
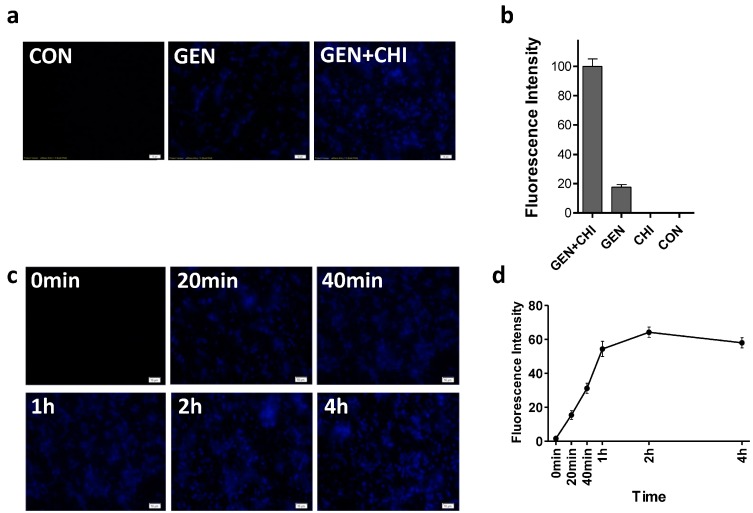
Chitosan-facilitated gentamicin accessibility into *L. monocytogenes* biofilms. *L. monocytogenes* biofilms were exposed to chitosan (200 µg/mL), gentamicin (5 µg/mL) and the mixture for 1 h (**a**,**b**) or different periods (**c**,**d**). Biofilms incubated with TSB were used as control. Gentamicin residing in biofilms was examined by Immunofluorescence. Immunoreactivity was quantified by using Image Pro Plus. Scale bars = 10 µm.

It has been suggested that positive charges make aminoglycoside antibiotics bind to negatively charged polymers in the biofilm matrix, which might be responsible for the slow penetration of antibiotics [18]. Since chitosan has been shown to penetrate biofilms due to the ability of cationic chitosan to disrupt negatively charged cell membranes as microbes settle on the surface [6,19], we attempted to see whether chitosan could make more gentamicin accessible into biofilms. Using a primary polyclonal antibody to gentamicin produced in rabbit and a second Dylight 405-conjugated goat anti-rabbit IgG, gentamicin residing in *L. monocytogenes* biofilms was visualized. Biofilms exposed to gentamicin alone exhibited a weak blue fluorescence (Figure 7a,b). In contrast, the intense blue fluorescence was observed in *L. monocytogenes* biofilms treated with the gentamicin/chitosan combination. The time-course of immunofluorescence indicated that gentamicin residing in *L. monocytogenes* biofilms increased very quickly in 1 h and then remained almost constant (Figure 7c,d).

**Figure 8:**
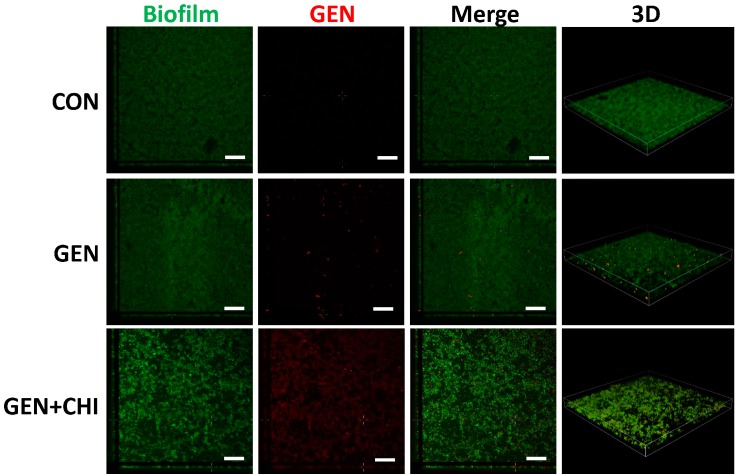
CLSM analysis of gentamicin penetration into biofilms. *L. monocytogenes* biofilms were exposed to chitosan (200 µg/mL), gentamicin (5 µg/mL) or the mixture for 1 h. The biofilm is depicted in green, while gentamicin is depicted in red. Scale bars indicates 50 µm.

To evaluate whether chitosan made gentamicin penetration into or attachment to *L. monocytogenes* biofilms, 5-(4,6-dichlorotriazinyl) aminofluorescein (5-DTAF, green fluorescence) was used to label bacteria and gentamicin was visualized with a primary polyclonal antibody followed by a second Dylight 594-conjugated goat anti-rabbit IgG (red fluorescence). 3D reconstruction from confocal microscopy indicated that chitosan enabled gentamicin penetration into *L. monocytogenes* biofilms, instead of simple attachment to the surface (Figure 8).

### 2.5. The Gentamicin/Chitosan Combination Was Able to Disperse Biofilms Built by Other Listeria Species

**Figure 9:**
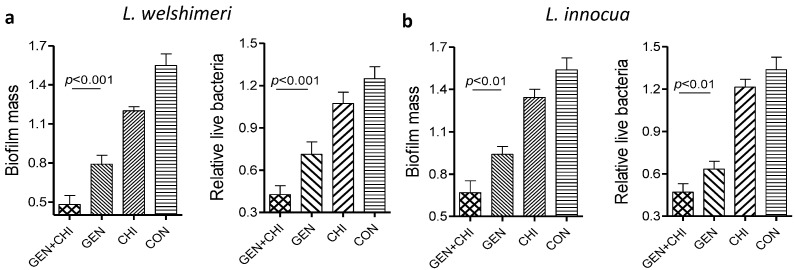
The chitosan-gentamicin combination was able to disperse biofilms built by other*Listeria* species. Biofilms built by *L. welshimeri* (**a**) or *L. innocua* (**b**) were performed for 24 h, and then exposed to chitosan (200 µg/mL), gentamicin (5 µg/mL) or the mixture for 24 h. Biofilm mass and viable cells were quantified. Error bars represent SD.

To see whether this combination was also effective in dispersal of biofilms built by other *Listeria* organisms, two *Listeria* species including *Listeria welshimeri* and *Listeria innocua* were tested. Quantification of biofilm biomass and cell viability demonstrated that the gentamicin/chitosan combination elicited a more pronounced effect than individual reagents did (Figure 9).

## 3. Experimental Section

### 3.1. Bacterial Strains

*L. monocytogenes* ATCC 15313 was a generous gift from Professor Xiaodong Xia, *L. welshimeri* (GIM1.232) and *L. innocua* (GIM1.365) were purchased from Guangdong Culture Collection Centre of Microbiology. The strains were cultivated in tryptic soy broth (TSB, Oxoid, Basingstoke, Hampshire, UK) at 37 °C for 12 h. Following incubation, dilution of the overnight culture with TSB matched to the 0.4 McFarland turbidity standard (approximately 10^8^ cfu/mL) was used for experimental procedures.

### 3.2. Chitosans & Antibiotics

Chitosans (CHI) with different molecular mass of 3000, 13,000, or 180,000 were purchased from Qingdao Yunzhou Bioengineering Co. Ltd. (Qingdao, China). The molecular weight of chitosan was determined on high performance gel permeation chromatography (HPGPC) as described [20]. All three chitosan had a similar *N*-deacetylation degree (88% ± 0.67% DD). Chitosan with lower *N*-deacetylation degree was generated by reaction of the original chitosan with acetic anhydride [21]. The DDA (degree of *N*-deacetylation) was measured by previous protocol [22]. All chitosan solutions were prepared by adding to TSB at a concentration of 1 mg·mL^−1^ (0.1%, *w*/*v*) [23].

Gentamicin (GEN), rifampicin (RFP), tetracycline (TET) and carbenicillin (CBC) were of USP (The United States Pharmacopeia) grade and purchased from Solarbio (Beijing, China). Stock solutions (10 mg·mL^−1^) were prepared in sterile water (gentamicin and carbenicillin) or dimethylsulfoxide (rifampicin and tetracycline) stored at 4 °C until use.

### 3.3. Biofilm Dispersal Experiments

100 μL bacterial TSB solutions (~10^8^ cfu) were seeded into 96-well polystyrene microtitre plates (Corning, New York, NY, USA) at 37 °C for 24 h to allow biofilm formation. The non-adhered cells were removed with pipette and the plate was washed three times using 100 μL 0.9% (*w*/*v*) NaCl. Then existing biofilms were incubated at 37 °C in 90 μL TSB supplemented with 10 μL antibiotic, chitosan or their mixture for different periods as indicated. Each treatment included 6 parallel wells. Biofilms incubated with TSB only were used as control. Biofilm mass (Crystal violet staining assay) [24] and viable cells (MTT (3-(4,5-dimethyl-thiazol-2-yl)-2,5-diphenyltetrazolium bromide) assay) [25] were evaluated. All experiments were performed 3–5 times. Error bars represent SD.

### 3.4. Fluorescence Microscopy

*L. monocytogenes* (~10^8^ cfu) was grown on glass coverslips at 37 °C for 24 h in 24-well plates supplemented with 1 mL of TSB to allow biofilm formation. The coverslips were washed to remove unattached cells and were treated with gentamicin, chitosan or their mixture for 6, 12 and 24 h at 37 °C. Existing biofilms were fixed using a 4% paraformaldehyde solution for 30 min at room temperature. After wash with 2 mL PBS, 5-(4,6-dichlorotriazinyl) aminofluorescein (5-DTAF) was added for 2 h at room temperature. The coverslips were washed 3 times with PBS. Images were acquired using the Olympus BX53 fluorescence microscope with a 100× (1.30) plan oil objective and processed by cellSens Entry software (Olympus, Tokyo, Japan).

### 3.5. Immunofluorescence

As above, biofilms on glass coverslips were fixed in 4% paraformaldehyde. After treatment with 0.25% Triton X-100 and blocking with 1% BSA in PBS, coverslips were incubated with a polyclonal antibody for gentamicin (rabbit anti-gentamicin ployclone, Abcam, Cambridge, MA, USA) at 4 °C overnight, and then incubated with a second Dylight 405-goat anti-rabbit IgG for fluorescence microscopy or Dylight 594-goat anti-rabbit IgG (Jackson Immuno Research Inc., West Grove, PA, USA) for confocal laser scanning microscope. Immunoreactivity was quantified by using Image Pro Plus (version 6.0, Media Cybernetics, Silver Spring, MD, USA). For confocal laser scanning microscopy (CLSM), imaging was performed with a Nikon A1R-A1 station (Nikon, Tokyo, Japan). Both image acquisition and subsequent manipulation were performed using Nikon NIS-Element software (Nikon, Tokyo, Japan).

### 3.6. Scanning Electron Microscopy (SEM)

Test samples were fixed in 2.5% glutaraldehyde at 4 °C for 24 h. Cells were rinsed with PBS three times for 10 min at each interval. The cultures were then dehydrated for 10 min in a gradient alcohol solution (50%, 70%, 80%, 90% and 100%). The specimen was left in 100% alcohol to prevent it from drying and mounted onto an aluminum stub with carbon tape, sputter-coated with gold (thickness of 10 nm) before examination. The scanning electron microscope (SEM) images were obtained by field emission scanning electron microscope (FESEM, S-4800, Hitachi, Tokyo, Japan).

### 3.7. Statistical Analysis

All graphs were made by GraphPad Prism 5.0. One way analysis of variance (ANOVA) was used to evaluate significant differences between different treatments and Newman–Keuls test was used for *post-hoc* test. *p* < 0.05 was considered significant differences.

## 4. Conclusions

In summary, our findings demonstrated that the combination of chitosan and gentamicin could effectively disrupt established *Listeria* biofilms at different stages. It seemed that chitosan with a moderate molecular mass (~13,000 Da) and high *N*-deacetylation degree (~88% DD) elicited an optimal anti-biofilm and bactericidal activity in this combination. Mechanistic insights indicated that the polycationic properties of chitosan enabled greater penetration of gentamicin into *Listeria* biofilms. This combinational strategy might be useful to combat *Listeria* biofilms when this specific antibiotic is recommended.

## References

[1] Lasa I. (2006). Towards the identification of the common features of bacterial biofilm development. Int. Microbiol..

[2] Romero R., Schaudinn C., Kusanovic J.P., Gorur A., Gotsch F., Webster P., Nhan-Chang C.-L., Erez O., Kim C.J., Espinoza J. (2008). Detection of a microbial biofilm in intraamniotic infection. Am. J. Obstet. Gynecol..

[3] Costerton J.W., Cheng K.J., Geesey G.G., Ladd T.I., Nickel J.C., Dasgupta M., Marrie T.J. (1987). Bacterial biofilms in nature and disease. Annu. Rev. Microbiol..

[4] Simões M., Simões L.C., Vieira M.J. (2010). A review of current and emergent biofilm control strategies. LWT.

[5] Ravi Kumar M.N.V. (2000). A review of chitin and chitosan applications. React. Funct. Polym..

[6] Rabea E.I., Badawy M.E.T., Stevens C.V., Smagghe G., Steurbaut W. (2003). Chitosan as antimicrobial agent: Applications and mode of action. Biomacromolecules.

[7] Zhang A., Mu H., Zhang W., Cui G., Zhu J., Duan J. (2013). Chitosan coupling makes microbial biofilms susceptible to antibiotics. Sci. Rep..

[8] Mu H., Zhang A., Zhang L., Niu H., Duan J. (2014). Inhibitory effects of chitosan in combination with antibiotics on Listeria monocytogenes biofilm. Food Control.

[9] Scallan E., Hoekstra R.M., Angulo F.J., Tauxe R.V., Widdowson M.-A., Roy S.L., Jones J.L., Griffin P.M. (2011). Foodborne illness acquired in the United States—Major pathogens. Emerg. Infect. Dis..

[10] Farber J.M., Peterkin P.I. (1991). *Listeria monocytogenes*, a food-borne pathogen. Microbiol. Rev..

[11] Low J.C., Donachie W. (1997). A review of *Listeria monocytogenes* and listeriosis. Vet. J..

[12] Pan Y., Breidt F., Kathariou S. (2006). Resistance of *Listeria monocytogenes* biofilms to sanitizing agents in a simulated food processing environment. Appl. Environ. Microbiol..

[13] Huang L., Dai T., Xuan Y., Tegos G.P., Hamblin M.R. (2011). Synergistic combination of chitosan acetate with nanoparticle silver as a topical antimicrobial: Efficacy against bacterial burn infections. Antimicrob. Agents Chemother..

[14] Schelegueda L.I., Gliemmo M.F., Campos C.A. (2012). Antimicrobial synergic effect of chitosan with sodium lactate, nisin or potassium sorbate against the bacterial flora of fish. J. Food Res..

[15] Tin S., Sakharkar K.R., Lim C.S., Sakharkar M.K. (2009). Activity of chitosans in combination with antibiotics in *Pseudomonas aeruginosa*. Int. J. Biol. Sci..

[16] Chavez de Paz L.E., Resin A., Howard K.A., Sutherland D.S., Wejse P.L. (2011). Antimicrobial effect of chitosan nanoparticles on streptococcus mutans biofilms. Appl. Environ. Microbiol..

[17] Shima S., Matsuoka H., Iwamoto T., Sakai H. (1984). Antimicrobial action of epsilon–poly-l-lysine. J. Antibiot..

[18] Stewart P.S., Costerton J.W. (2001). Antibiotic resistance of bacteria in biofilms. Lancet.

[19] Carlson R.P., Taffs R., Davison W.M., Stewart P.S. (2008). Anti-biofilm properties of chitosan-coated surfaces. J. Biomater. Sci. Polym. Ed..

[20] Zhang L., Zhang W., Wang Q., Wang D., Dong D., Mu H., Ye X.-S., Duan J. (2015). Purification, antioxidant and immunological activities of polysaccharides from *Actinidia Chinensis* roots. Int. J. Biol. Macromol..

[21] Kubota N., Tatsumoto N., Sano T., Toya K. (2000). A simple preparation of half *N*-acetylated chitosan highly soluble in water and aqueous organic solvents. Carbohydr. Res..

[22] Muzzarelli R.A.A., Rocchetti R. (1985). Determination of the degree of acetylation of chitosans by first derivative ultraviolet spectrophotometry. Carbohydr. Polym..

[23] Orgaz B., Lobete M.M., Puga C.H., San Jose C. (2011). Effectiveness of chitosan against mature biofilms formed by food related bacteria. Int. J. Mol. Sci..

[24] Djordjevic D., Wiedmann M., McLandsborough L.A. (2002). Microtiter plate assay for assessment of *Listeria monocytogenes* biofilm formation. Appl. Environ. Microbiol..

[25] Schillaci D., Arizza V., Dayton T., Camarda L., Stefano V.D. (2008). *In vitro a*nti-biofilm activity of *Boswellia* spp. oleogum resin essential oils. Lett. Appl. Microbiol..

